# Recurrent massive hematoma after trivial trauma in Ehlers–Danlos syndrome: a case report highlighting diagnostic urgency and surgical challenges

**DOI:** 10.1093/jscr/rjag367

**Published:** 2026-05-16

**Authors:** Mohamed B Ahmed, Hillal Bhat, Yawar Nissar, Fatima S Al-Mohannadi, Haithem E Babiker

**Affiliations:** Plastic Surgery Department, Hamad General Hospital, Hamad Medical Corporation, Doha, Qatar; College of Medicine, QU Health, Qatar University, Doha, Qatar; Division of Plastic and Craniofacial Surgery, Sidra Medicine, Doha, Qatar; Division of Plastic and Craniofacial Surgery, Sidra Medicine, Doha, Qatar; Plastic Surgery Department, Hamad General Hospital, Hamad Medical Corporation, Doha, Qatar; Division of Plastic and Craniofacial Surgery, Sidra Medicine, Doha, Qatar; Weill Cornell Medicine, Doha, Qatar

**Keywords:** Ehlers–Danlos syndrome, musculocontractural EDS, traumatic hematoma, pediatric reconstructive surgery

## Abstract

Musculocontractural Ehlers–Danlos syndrome (mcEDS) is a rare connective tissue disorder characterized by abnormal collagen structure, tissue fragility, and predisposition to disproportionate hematoma formation following trivial trauma. Large expanding hematomas may lead to tissue necrosis, thereby requiring urgent decision-making and rapid intervention. We report a case of a 10-year-old boy with mcEDS and Dandy–Walker syndrome who presented after a minor fall with rapidly progressive swelling of the right lower limb and a history of recurrent post-traumatic hematomas. Clinical deterioration prompted urgent exploration, revealing a large, organized hematoma, and friable devitalized tissues. Extensive debridement, staged wound care, and split-thickness skin grafting were performed. Microbiology was negative, confirming a traumatic hematoma with secondary soft-tissue compromise related to the connective tissue disorder. Minor trauma in patients with mcEDS may cause severe limb-threatening hematomas with tissue necrosis. Early recognition and prompt surgical intervention are essential to prevent ischemic injury and improve reconstructive outcomes.

## Introduction

Ehlers–Danlos syndrome (EDS) is a group of inherited connective tissue conditions with diverse genetic causes and isconsidered a rare disorder, with a reported prevalence of ~1 per 5000 individuals [1]. Most identified mutations involve genes encoding fibrillar collagens, collagen-processing proteins, or enzymes for glycosaminoglycan biosynthesis, all essential for maintaining extracellular matrix structural integrity across tissues and organs [2]. Consequently, common clinical manifestations include hypermobile joints, fragile soft tissues, and increased skin elasticity [3].

An even rarer subtype of EDS is musculocontractural EDS (mcEDS), which results from biallelic loss of function variants in either the CHST14 gene (carbohydrate sulfotransferase 14/dermatan 4-O-sulfotransferase 1) or the DSE gene (dermatan sulfate epimerase), which encodes enzymes involved in dermatan sulfate synthesis [3]. It is characterized by multiple congenital abnormalities and progressive manifestations of connective-tissue fragility, such as easily bruised skin, abnormal scarring, joint dislocations, skeletal deformities, spontaneous pneumothorax, large subcutaneous hematomas, and gastrointestinal perforation [4]. We report a pediatric mcEDS patient who developed a rapidly expanding lower-limb hematoma after minor trauma, causing severe soft-tissue compromise and requiring urgent surgical intervention.

## Case presentation

A 10-year-old boy presented to the emergency department with progressive swelling, extensive bruising, and a superficial wound over the right ankle following a minor fall at home 4 days earlier. His symptoms gradually worsened with increasing pain and circumferential swelling of the right lower extremity, prompting emergency evaluation. The patient had a known diagnosis of Dandy–Walker syndrome with developmental delay and mcEDS, confirmed by genetic testing identifying a pathogenic mutation in the CHST14 gene. His condition was associated with connective tissue fragility, abnormal wound healing, and musculoskeletal abnormalities, including bilateral complex clubfoot with prior surgical correction.

The patient’s medical history was notable for recurrent, disproportionate hematoma formation following trivial trauma requiring operative management. These included evacuation of a large scalp and subgaleal hematoma by neurosurgery and plastic surgery teams, followed 2 years later by evacuation of another massive scalp and forehead hematoma during which ~1 L of blood was removed. These episodes occurred in the absence of significant trauma or identifiable coagulopathy.

**Figure 1:**
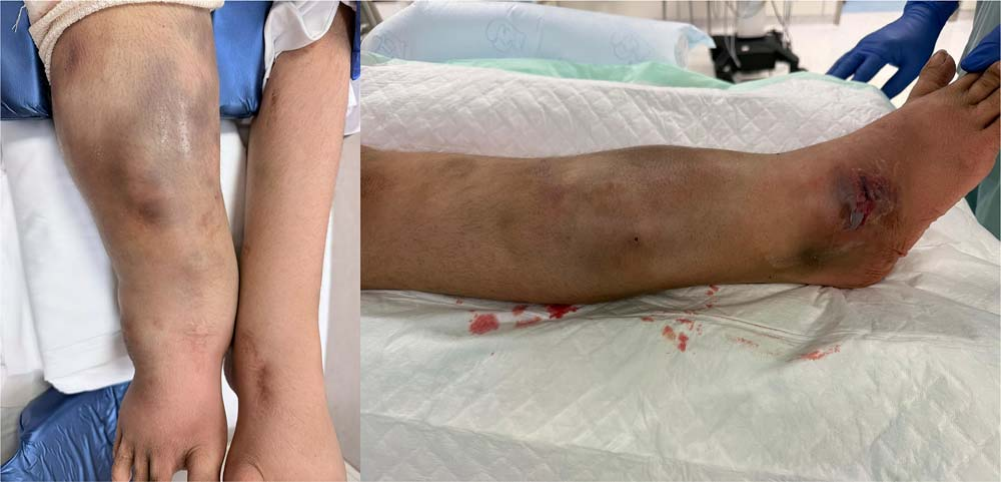
Preoperative presentation. Preoperative clinical photographs demonstrating extensive right lower-limb swelling and skin discoloration consistent with a large post-traumatic hematoma following minor injury. The appearance reflects significant soft-tissue compromise in the setting of underlying connective tissue fragility.

On presentation, the patient was alert but tachycardic. Physical examination demonstrated extensive circumferential ecchymosis and tense swelling of the right lower extremity extending from below the knee to the ankle, with compromised overlying skin characterized by discoloration and early blistering (Fig. 1). The posterior compartment remained relatively soft, while the anterior and medial compartments were markedly tense. The contralateral limb was normal. Plain radiographs showed no osseous injury.

Given the progressive swelling, skin compromise, and concern for an expanding hematoma, we proceeded with emergent surgical exploration to preserve tissue viability. Intraoperatively, a large circumferential deep hematoma totaling ~1 L was identified, along with extensive nonviable skin, subcutaneous tissue, fascia, and focal muscle necrosis. The underlying musculature was well perfused. Wide evacuation of the hematoma and circumferential debridement were performed, achieving hemostasis. The wound was extensively irrigated, temporarily packed, and dressed.

**Figure 2:**
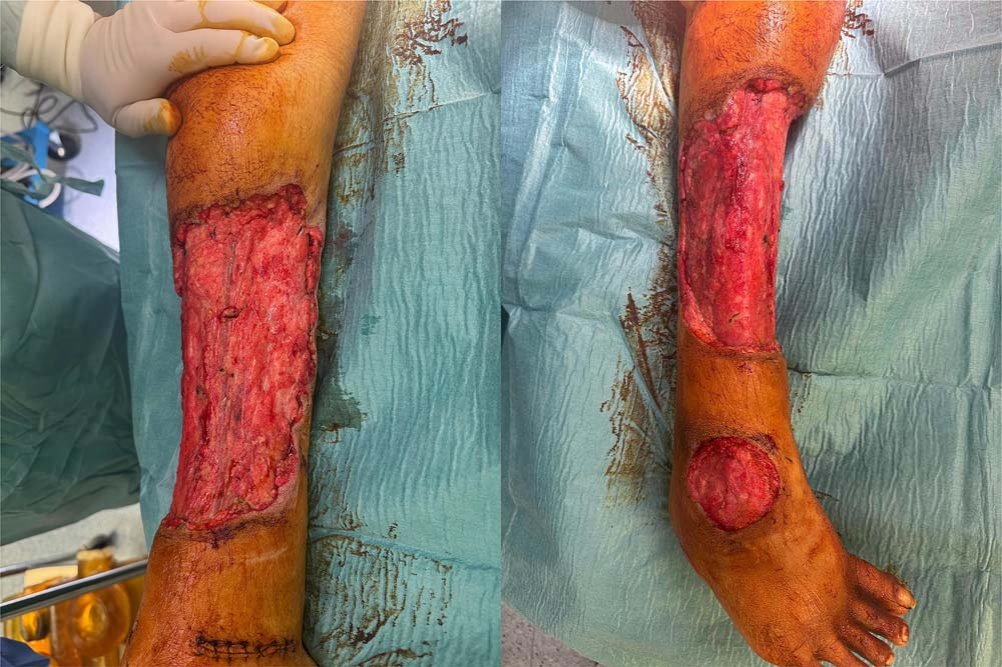
Intraoperative findings. Intraoperative photographs showing the lower limb after multiple surgical debridements and serial vacuum-assisted closure dressing changes, at the stage when the wound was deemed suitable for split-thickness skin grafting.

Postoperatively, the patient was admitted to the intensive care unit for monitoring, received broad-spectrum intravenous antibiotics, and required transfusion of two units of packed red blood cells. Histopathology demonstrated skin necrosis with acute and chronic inflammation. Over subsequent days, the patient underwent planned second-look operations, serial debridement, and negative-pressure wound therapy (Fig. 2).

**Figure 3:**
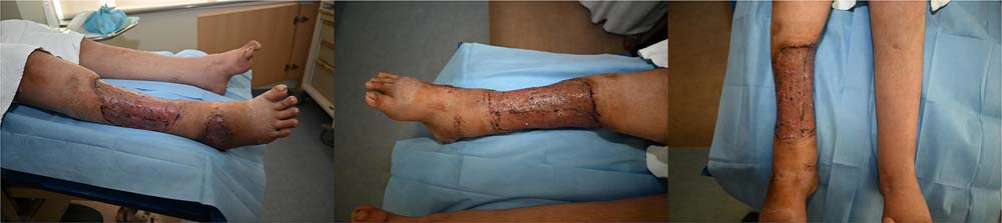
Postoperative course. Postoperative photographs 23 days following staged wound management and split-thickness skin grafting, demonstrating satisfactory graft take and progressive wound healing of the right lower limb.

After development of healthy granulation tissue, split-thickness skin grafting was performed 12 days after presentation. Follow-up examination confirmed satisfactory graft condition. Outpatient review 10 days later demonstrated complete graft take without infection and satisfactory donor-site healing. The patient was discharged after 17 days of hospitalization in stable condition and continued to recover without complications (Fig. 3). Early mobilization was encouraged, and full weight-bearing ambulation was achieved on postoperative day 1.

## Discussion

mcEDS is a rare connective tissue disorder caused by CHST14 mutations, characterized by tissue fragility, impaired collagen fibril assembly, extracellular matrix disorganization, and vascular vulnerability [5]. Patients may develop disproportionate hematomas after minor trauma, a potentially limb-threatening complication rarely described in soft-tissue injury management [3].

A prior report described a 6-year-old boy with mcEDS who developed a giant lower limb hematoma after minor trauma, resulting in hemorrhage and shock managed conservatively with compression and tranexamic acid [5]. In contrast, our patient developed rapid hematoma expansion with soft tissue compromise and threatened skin viability, requiring urgent surgical evacuation, serial debridement, and staged reconstruction. This comparison highlights the clinical heterogeneity of hematoma complications in mcEDS and the need for individualized management based on disease progression and tissue compromise. A recent report described a 26-year-old man with mcEDS who developed a massive subcutaneous hematoma after minor trauma with arterial bleeding extending from the lumbar region toward the scapula, leading to hypovolemic shock and requiring emergency evacuation [6]. Initially stable and managed conservatively, hematoma expansion later required surgery. This highlights the variable hemorrhagic presentations of mcEDS and the need for individualized management with timely surgical intervention when deterioration occurs.

This case highlights the importance of early surgical evaluation and intervention. Despite initial hemodynamic stability and normal imaging, circumferential swelling, skin compromise, and pain required urgent exploration. Delayed management of EDS-related hemorrhage may cause compartment syndrome, infection, sepsis, or death, including a reported fatal septicemia from untreated scalp hemorrhage [3, 7, 8]. Operative findings revealed an extensive circumferential hematoma with nonviable skin, subcutaneous tissue, fascia, and muscle—an appearance rarely reported in EDS hematomas. Differentiating hematoma-related necrosis can be challenging, making exploration essential. A staged strategy—hematoma evacuation, serial debridement, negative-pressure therapy, and delayed grafting—enabled limb salvage, with multidisciplinary collaboration critical for optimal outcomes [9].

This case highlights key lessons: disproportionate hematomas after minor trauma should raise suspicion for connective tissue disorders. In mcEDS, soft tissue injuries may progress rapidly. Early surgical intervention is essential when expanding hematoma or skin compromise occurs, as delayed treatment risks tissue loss. Vigilance, timely decision-making, and staged reconstruction are crucial for optimal outcomes.

## Conclusion

This case highlights severe bleeding in mcEDS, where minor trauma may cause massive hematomas and tissue necrosis. Early recognition, multidisciplinary management, and prompt surgical intervention are essential to prevent tissue compromise.

## Data Availability

All available data is presented within the case report. Additional and follow up data may be requested from the corresponding author upon reasonable request.
